# 124例成人噬血细胞综合征临床预后分析：一项淮海淋巴瘤协作组多中心回顾性研究

**DOI:** 10.3760/cma.j.issn.0253-2727.2021.10.002

**Published:** 2021-10

**Authors:** 子园 沈, 晨露 贺, 倩 孙, 硕 张, 灵灵 胡, 沁华 刘, 颢 张, 鑫 刘, 雨青 苗, 伟英 顾, 飞 王, 春玲 王, 玉叶 史, 玲 王, 俊 焦, 静静 叶, 林艳 徐, 冬梅 闫, 振宇 李, 英良 金, 水平 黄, 开林 徐, 威 桑

**Affiliations:** 1 徐州医科大学公共卫生学院流行病与卫生统计学系 221004 Department of Epidemiology and Health Statistics, School of Public Health, Xuzhou Medical University, Xuzhou 221004, China; 2 徐州医科大学附属医院血液科 221002 Department of Hematology, Affiliated Hospital of Xuzhou Medical University, Xuzhou 221002, China; 3 安徽医科大学附属第一医院血液科，合肥 230022 Department of Hematology, The First Affiliated Hospital of Anhui Medical University, Hefei 230022, China; 4 济宁医学院附属医院血液科 272000 Department of Hematology, The Affiliated Hospital of Jining Medical University, Jining 272000, China; 5 盐城市第一人民医院血液科 224001 Department of Hematology, Yancheng First People's Hospital, Yancheng 224001, China; 6 常州市第一人民医院血液科 213003 Department of Hematology, The First People's Hospital of Changzhou, Changzhou 213003, China; 7 淮安市第一人民医院血液科 223000 Department of Hematology, The First People's Hospital of Huaian, Huaian 223300, China; 8 泰安市中心医院血液科 271000 Department of Hematology, Taian Central Hospital, Taian 271000, China; 9 山东大学齐鲁医院血液科，济南 250012 Department of Hematology, Qilu Hospital of Shandong University, Jinan 250012, China

**Keywords:** 成人, 噬血细胞综合征, 预后分析, Adult, Hemophagocytic lymphohistiocytosis, Prognostic analysis

## Abstract

**目的:**

基于多中心数据，分析成人噬血细胞综合征（HLH）预后的影响因素。

**方法:**

收集2014年3月至2020年7月淮海淋巴瘤协作组8个医疗中心确诊的124例成人HLH患者的临床资料。基于Maxstat算法、X-Tile软件和限制立方样条获取连续变量的最佳截断值。采用Cox比例风险回归模型构建成人HLH风险预测模型并通过列线图实现模型的可视化，采用Bootstrap重抽样的方法进行验证，使用一致性指数（C-index）和校正曲线验证列线图，检查预测精度。采用Kaplan-Meier法分析计算生存率并绘制生存曲线，组间比较使用Log-rank检验。

**结果:**

124例患者的中位年龄为55（18～84）岁，男性61例（49.19％）。最常见的病因是感染，血清铁蛋白增高者110例（88.71％），肝脾肿大者57例（45.97％）。124例患者中77例（62.10％）死亡，患者的中位生存期为7.07个月，单因素分析结果表明成人HLH的预后受性别、年龄、纤维蛋白原、血肌酐、ALT和白蛋白的影响（*P*<0.05）。多因素分析结果表明性别、PLT、白蛋白、ALT和治疗方案是预后的独立影响因素，基于以上5个危险因素建立列线图预测模型，模型的C-index为0.739，校准图显示HLH的观测值和预测值之间有较好的一致性。

**结论:**

成人HLH的预后受多方面因素影响，性别、PLT、白蛋白、ALT和治疗方案是独立危险因素，基于上述危险因素建立的列线图为临床医师评估成人HLH预后提供了一个可视化工具。

## Abstract (en)

**Objective:**

Factors influencing the prognosis of hemophagocytic lymphohistiocytosis (HLH) in adults were analyzed based on multicentric data.

**Methods:**

Clinical data of 124 adult patients with HLH diagnosed in eight medical centers in the Huaihai Lymphoma Working Group from March 2014to July 2020 were collected. The optimal truncation value of continuous variables was obtained based on the Maxstat algorithm, X-Tile software, and restricted cubic spline. Cox proportional risk regression model was used to construct the adult HLH risk prediction model, and the visualization of the model was realized through the histogram. The bootstrap resampling method was used to verify the model, C-index and calibration curve was used to verify the histogram, and the prediction accuracy was checked. Kaplan-Meier analysis was used to calculate the survival rate and draw the survival curve. Furthermore, the differences between groups were tested by log-rank.

**Results:**

The median age of the 124 patients was 55 (18–84) years, including 61 (49.19％) males. The most common etiology was infection. Serum ferritin increased in 110 cases (88.71％), hepatosplenomegaly in 57 cases (45.97％). Of the 124 patients, 77 (62.10％) died, and the median survival time of the patients was 7.07 months. Univariate results showed that the prognosis of adult HLH was influenced by sex, age, fibrinogen, serum creatinine, alanine aminotransferase, and albumin (*P*<0.05). The results of multivariate analysis showed that gender, platelet, albumin, alanine aminotransferase, and treatment regimens were independent influencing factors for prognosis. Based on the above five risk factors, the prediction model of the histogram was established, and the C-index of the model was 0.739. Finally, the calibration chart showed good consistency between the observed and predicted values of HLH.

**Conclusion:**

The prognosis of the adult hemophagocytic syndrome is influenced by many factors. Gender, platelet, albumin, alanine aminotransferase, and treatment regimens are independent risk factors. Therefore, the established histogram provides a visual tool for clinicians to evaluate the prognosis of adult HLH.

噬血细胞综合征又称噬血细胞性淋巴组织细胞增生症（HLH），其特征是免疫细胞过度刺激导致全身性炎症和多脏器功能衰竭[1]。根据诱因不同，一般分为原发性（遗传性）和继发性（获得性）两类。临床表现以持续性发热、肝脾肿大和全血细胞减少为主。本研究回顾性收集淮海淋巴瘤协作组8个医疗中心124例成人HLH患者的临床资料，分析成人HLH的预后影响因素，为患者的预后判断提供参考依据。

## 病例与方法

1. 病例：淮海淋巴瘤协作组（HHLWG）成立于2017年11月，包括中国淮海经济区的18个医疗中心。本研究纳入2014年3月至2020年7月淮海淋巴瘤协作组8个医疗中心确诊为HLH的成年（≥18岁）患者124例，收集患者的临床资料。8个医疗中心分别为徐州医科大学附属医院（54例）、济宁医学院附属医院（20例）、山东齐鲁医院（18例）、盐城市第一人民医院（11例）、安徽医科大学附属第一医院（7例）、淮安市第一人民医院（7例）、泰安市中心医院（5例）、常州市第一人民医院（2例）。

2. 诊断标准：HLH的诊断参照国际组织细胞协会HLH-2004诊断标准，符合以下8条标准中的5条即可诊断：①发热；②脾大；③血细胞减少，累及外周血两系或三系；④高甘油三酯血症和（或）低纤维蛋白原血症，空腹甘油三酯（TG）≥3.0 mmol/L，纤维蛋白原（FIB）≤1.5 g/L；⑤血清铁蛋白（Fer）升高（≥500 µg/L）；⑥可溶性白细胞介素-2受体（sCD25）≥2 400 U/ml；⑦NK细胞活性降低或缺乏；⑧骨髓、脾脏或淋巴结活检有吞噬血细胞现象，无恶性肿瘤性疾病证据。

3. 研究方法：筛选符合诊断标准的成人HLH患者，收集患者确诊HLH时的年龄、病因、Fer、TG、FIB、LDH、白蛋白（ALB）、血肌酐（Cr）、ALT、HGB、PLT和淋巴细胞计数（LYR）等指标。通过胸腹部CT平扫、B超明确患者有无肝、脾、浅表淋巴结肿大。

4. 随访：通过查阅患者电子病历及纸质病历确认患者住院治疗情况，对患者进行电话随访，随访时间截至2021年1月。总生存（OS）期定义为患者确诊HLH至因任何原因死亡或随访截止的时间间隔。

5. 统计学处理：连续变量分布采用Shapiro-Wilk检验进行正态性分析，计量资料的组间比较采用Mann-Whitney *U*检验，多组比较采用Kruskal-Wallis检验，计数资料的组间比较采用Pearson *χ*^2^检验。连续变量采用基于Maxstat统计量、限制立方样条和X-Tile的方法，以确定连续变量的最佳截断值[2]。预后分析采用Kaplan-Meier曲线，组间比较采用Log-rank检验，采用Cox比例风险模型进行单因素、多因素分析，基于多因素回归结果建立并检验列线图，应用Bootstrap重抽样（B＝1000）计算C指数（C-index），校准曲线评价模型性能，*P*<0.05为差异有统计学意义。所有统计学分析采用SPSS 19.0、R软件和X-Tile软件。

## 结果

1. 患者基线资料：124例成人HLH患者中男61例（49.2％），女63例（50.8％），中位年龄55（18～84）岁。124例患者中105例（84.68％）发热，多为持续性不规则高热，110例（88.71％）血清Fer增高，57例（45.97％）肝脾肿大，86例（69.35％）患者ALT超过正常参考值上限，40例（32.26％）患者FIB低于正常值。124例患者中77例（62.10％）死亡，患者的中位OS时间为7.07（0.03～71.73）个月。

2. 病因分布和生存分析：感染相关HLH患者64例（52％），其中EB病毒（EBV）感染35例（28％）；淋巴瘤相关HLH患者23例（19％）；EBV感染合并淋巴瘤患者7例（6％）；风湿性疾病患者3例（2％）；其他恶性血液病/肿瘤患者6例（4％）；病因不明者21例（17％）。对各病因组生存曲线进行分析，总体上各组差异无统计学意义（*P*＝0.061）（图1），进一步行组间分析显示，EBV感染组患者与其他感染组患者的差异有统计学意义（*χ*^2^＝6.297，*P*＝0.012），感染组患者和风湿性疾病患者的OS率均较病因不明组患者高（*χ*^2^值分别为6.297、3.874，*P*值分别为0.012、0.049）。淋巴瘤合并EBV感染患者与淋巴瘤不合并EBV感染患者相比，OS率的差异无统计学意义（*χ*^2^＝0.046，*P*＝0.829）。淋巴瘤患者的OS率与单纯EBV感染患者相比，差异无统计学意义（*χ*^2^＝0.452，*P*＝0.502）。

**图1:**
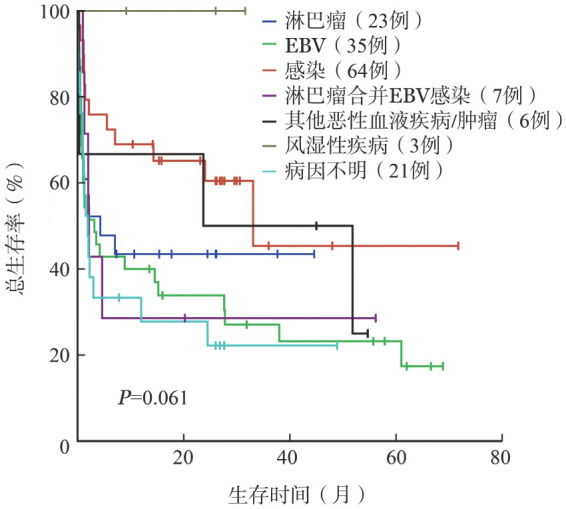
124例不同病因噬血细胞综合征患者的总生存曲线

124例患者中17例采用HLH-2004治疗方案，具体为：初始治疗（1～8周）：地塞米松10 mg·m^−2^·d^−1^×2周，5 mg·m^−2^·d^−1^×2周，2.5 mg·m^−2^·d^−1^×2周，1.25 mg·m^−2^·d^−1^×1周，减停1周；环孢素A 1.5～2.5 mg·m^−2^·d^−1^，血药浓度控制在200～400 µg/L；依托泊苷150 mg/m^2^，2次/周×2周；依托泊苷150 mg/m^2^，1次/周×6周。维持治疗（共32周）：地塞米松10 mg·m^−2^·d^−1^，第10周开始，隔周应用，每次连用3 d；依托泊苷150 mg·m^−2^·d^−1^，第9周开始，隔周应用一次；环孢素A剂量及用法同前。18例患者采用HLH-94方案，具体为：初始治疗（1～8周）：地塞米松，10 mg·m^−2^·d^−1^×2周，5 mg·m^−2^·d^−1^×2周，2.5 mg·m^−2^·d^−1^×2周，1.25 mg·m^−2^ ·d^−1^×1周，减停1周；依托泊苷150 mg/m^2^，2次/周×2周，1次/周×6周；有神经症状者或脑脊液异常者，甲氨蝶呤鞘内注射，第3周开始，每周1次，共4次。维持治疗（9～52周）：地塞米松10 mg·m^−2^·d^−1^×3 d/2周；依托泊苷150 mg/m^2^，1次/2周；环孢素A 6 mg·m^−2^·d^−1^，口服，使血药浓度维持在200 mg/L左右，直至52周，52周后行造血干细胞移植。12例采用CHOP/CHOP-like方案，具体为：环磷酰胺750 mg/m^2^，第1天，静脉注射；阿霉素50 mg/m^2^，第1天，静脉注射；长春新碱1.4 mg/m^2^，第1天，静脉注射；泼尼松100 mg·m^−2^·d^−1^，口服，第1～5天。4例采用DEP方案，具体为：脂质体阿霉素25 mg/m^2^，第1天；依托泊苷100 mg/m^2^，每周1次；甲泼尼龙，15 mg/kg第1～3天，2 mg/kg第4～6天，1 mg/kg第7～10天，0.75 mg/kg第11～14天，0.5 mg/kg第15～21天，0.4 mg/kg第22～28天。采用HLH-94方案、HLH-2004方案、DEP方案和CHOP/CHOP-like方案患者的1年OS率分别为35.3％、33.3％、75.0％和75.0％。对HLH-2004方案、HLH-94方案、DEP方案和CHOP/CHOP-like方案组的生存曲线进行分析，总体上差异有统计学意义（*P*＝0.035）（图2），HLH-94方案与CHOP/CHOP-like方案组患者OS率的差异有统计学意义（*P*＝0.006），CHOP/CHOP-like方案组的OS优于HLH-94方案组。HLH-2004方案组与HLH-94方案组OS率的差异无统计学意义（*P*＝0.542）。

**图2:**
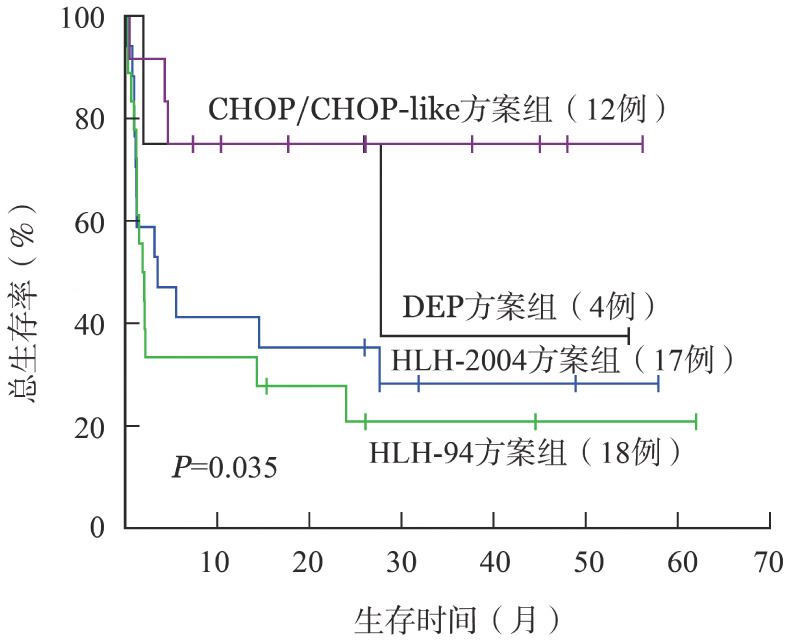
124例不同治疗方案噬血细胞综合征患者的总生存曲线

对存活组与死亡组患者的临床特征及实验室检查结果进行分析，Mann-Whitney *U*检验结果表明存活组与死亡组年龄、FIB、PLT和ALB的差异有统计学意义，Fer、TG、LDH、Cr的差异无统计学意义（*P*值均>0.05）（表1）。Pearson *χ*^2^结果表明存活组与死亡组病因（*P*＝0.014）和性别（*P*＝0.003）的差异有统计学意义，发热（*P*＝0.162）和肝脾肿大（*P*＝0.181）的差异无统计学意义。

**表1:** 存活组与死亡组噬血细胞综合征患者年龄及实验室检查结果比较［*M*（*P*_25_，*P*_75_）］

因素	存活组 (47例)	死亡组 (77例)	*Z*值	*P*值
年龄 (岁)	53.0 (26.0,65.0)	54.5 (39.8,62.3)	−1.984	0.047
铁蛋白 (µg/L)	2000.0 (843.0,10681.0)	2000.0 (9000.0,4457.0)	−0.533	0.594
甘油三酯 (mmol/L)	1.8 (1.3,2.9)	2.0 (1.4,2.8)	−0.322	0.747
纤维蛋白原 (g/L)	2.5 (1.7,3.7)	1.8 (1.2,3.9)	−2.212	0.027
LDH (U/L)	767.0 (407.0,1591.0)	835.5 (393.3,1443.0)	−0.626	0.531
血肌酐 (µmol/L)	52.0 (41.1,68.0)	58.0 (44.8,70.6)	−1.268	0.205
ALT (U/L)	45.0 (23.0,76.0)	50.0 (28.0,122.3)	−1.389	0.165
HGB (g/L)	105.0 (80.0,116.0)	93.0 (69.0,115.0)	−1.409	0.159
PLT (×10^9^/L)	82.0 (46.0,124.0)	48.5 (25.8,73.8)	−3.374	0.001
淋巴细胞计数 (×10^9^/L)	22.3 (14.4,44.0)	25.9 (15.8,38.0)	−0.458	0.647
白蛋白 (g/L)	33.6 (27.9,38.2)	31.8 (27.1,35.7)	−2.016	0.044

比较肿瘤、感染、风湿性疾病相关HLH患者确诊时的实验室检查，发现三组HLH患者Fer、TG、FIB、LDH、Cr、ALT、PLT、HGB、ALB的差异均无统计学意义（*P*值均>0.05）（表2），LYR的组间差异有统计学意义，进一步组间分析表明，感染相关HLH组的LYR高于肿瘤相关HLH组和风湿性疾病相关HLH组。

**表2:** 肿瘤、感染、风湿性疾病相关噬血细胞综合征（HLH）患者确诊时实验室检查结果比较［*M*（*P*_25_，*P*_75_）］

因素	肿瘤相关HLH (29例)	感染相关HLH (64例)	风湿性疾病相关HLH (3例)	*H*值	*P*值
铁蛋白 (µg/L)	929.3 (1995.3,7294.8)	910.3 (1990.5，4724.2)	1066.6 (1290.3，11527.0)	0.250	0.880
甘油三酯 (mmol/L)	1.6 (2.2,2.7)	1.3 (2.0，2.8)	2.6 (3.5，4.2)	0.050	0.980
纤维蛋白原 (g/L)	1.2 (2.0,3.4)	1.3 (2.1，3.5)	1.8 (2.6，3.1)	2.363	0.307
LDH (U/L)	334.0 (612.0,1129.0)	394.5 (653.4,1419.0)	907.0 (1423.0,1507.0)	2.966	0.227
血肌酐 (µmol/L)	47.0 (62.3,69.5)	43.4 (55.5,63.5)	59.8 (68.0,79.5)	1.837	0.399
ALT (U/L)	25.5 (66.0,138.4)	35.5 (63.8,156.4)	126.5 (208.0,865.2)	1.344	0.511
PLT (×10^9^/L)	27.0 (60.0,87.5)	34.0 (62.0,107.5)	78.0 (102.0,117.0)	2.046	0.360
HGB (g/L)	77.0 (96.5,112.0)	81.5 (93.5，113.0)	105.0 (112.0,124.5)	0.620	0.733
淋巴细胞计数 (×10^9^/L)	10.5 (15.8,27.1)	15.6 (25.4,39.1)	13.8 (16.4,21.2)	9.241	0.010
白蛋白 (g/L)	27.8 (31.2,34.3)	27.0 (31.4,35.7)	31.8 (37.3,38.0)	1.155	0.561

3. 最佳截断值的确定：基于Maxstat统计量计算出年龄的最佳截断值为41岁，FIB的最佳截断值为2.36 g/L，LDH的最佳截断值为228.3 U/L，Cr的最佳截断值为56.4 µmol/L，ALT的最佳截断值为76 U/L，ALB的最佳截断值为31.8 g/L。通过限制立方样条计算出PLT的最佳截断值为58.5×10^9^/L。使用X-Tile软件计算出HGB的最佳截断值为104 g/L，Fer的最佳截断值为775 µg/L和4 076 µg/L，淋巴细胞百分比的最佳截断值为27.2％和48.1％，身体质量指数的最佳截断值为18.0 kg/m^2^和20.6 kg/m^2^，TG的最佳截断值为1.1 mmol/L和2.1 mmol/L。

4. 单因素、多因素分析：单因素分析表明成人HLH的预后受以下因素影响：性别、年龄、PLT、FIB、ALT、ALB和Cr（*P*值均<0.05）（表3）。将上述因素、治疗方案和病因纳入Cox模型采用向前逐步法进行多因素分析，结果表明性别、PLT、ALB、ALT和治疗方案是预后的独立影响因素，LDH对预后存在边界影响（表3）。

**表3:** 124例噬血细胞综合征患者预后影响因素的单因素与多因素分析

因素	单因素分析		多因素分析	
	*HR*（95％ *CI*）	*P*值	*HR*（95％ *CI*）	*P*值
性别（男）	0.415（0.262～0.658）	<0.001	0.417（0.257～0.677）	<0.001
PLT（< 58.5×10^9^/L）	0.470（0.296～0.747）	0.001	0.548（0.342～0.877）	0.012
白蛋白（< 31.8g/L）	0.507（0.320～0.804）	0.004	0.573（0.352～0.934）	0.025
ALT（< 76U/L）	1.795（1.143～2.817）	0.011	1.727（1.062～2.900）	0.028
治疗（HLH-2004方案）	0.903（0.805～1.012）	0.081	0.878（0.780～0.988）	0.031
LDH（< 228.3U/L）	2.430（0.887～6.655）	0.084	2.419（0.872～6.710）	0.090
病因（淋巴瘤）	1.030（0.992～1.156）	0.617	–	–
纤维蛋白原（< 2.36g/L）	0.556（0.348～0.889）	0.014	–	–
血肌酐（< 56.4µmol/L）	1.730（1.092～2.741）	0.020	–	–
年龄（< 41岁）	1.877（1.080～3.260）	0.025	–	–

注：–：未进行多因素分析

5. 成人HLH列线图的构建及验证：本研究基于性别、PLT、ALB、ALT和治疗方案五个独立危险因素建立预测成人HLH预后的列线图模型（图3），并对该模型使用校准图进行验证（图4），预测值同实测值基本一致，说明本研究的列线图预测模型具有较好的预测能力，同时使用Bootstrap重抽样法对列线图模型进行验证，C-index为0.739。

**图3:**
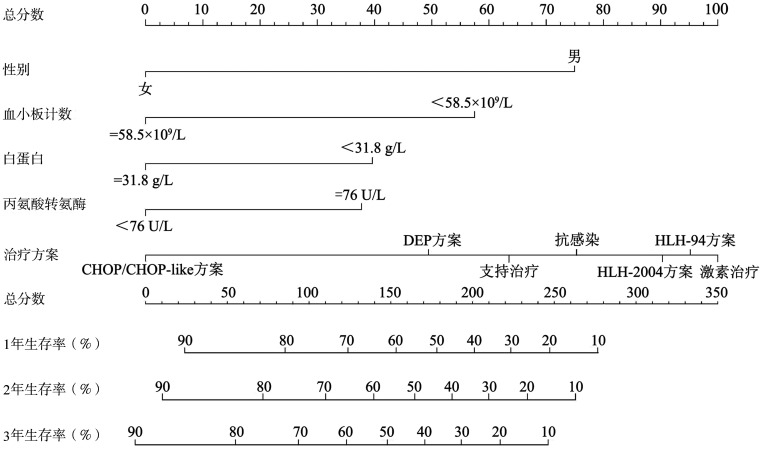
124例成人噬血细胞综合征患者预后的列线图模型

**图4:**
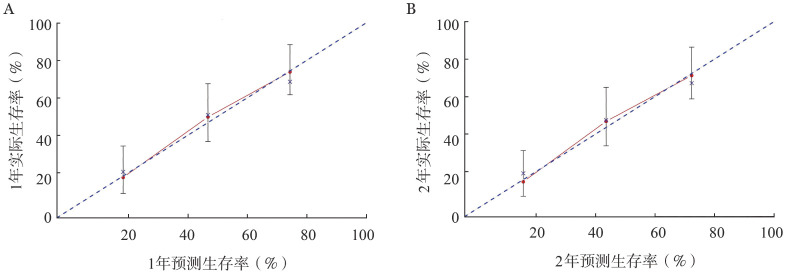
124例成人噬血细胞综合征患者预后列线图模型1年（A）、2年（B）校准图

## 讨论

HLH是一种以过度炎症反应为特征的临床综合征，其病因多样，EBV感染、淋巴瘤、自身免疫性疾病均是诱发HLH的常见因素。HLH在临床上呈高度侵袭性，未经治疗的原发性HLH患者的中位OS期仅2个月。HLH异质性较强，由于缺乏有效的预后分层模型，在临床治疗中缺乏成熟的个体化治疗策略。本研究通过回顾性分析124例淮海淋巴瘤协作组多中心成人HLH的临床数据，探索成人HLH的预后影响因素。

在本研究中我们发现，成人HLH的诱因以感染为主，其中EBV相关HLH最常见，其次是淋巴瘤、风湿性疾病和其他恶性血液病，EBV感染和淋巴瘤仍然是成人HLH最常见的诱因，与既往的研究结果基本一致[3]。淋巴瘤相关HLH可分为淋巴瘤诱导的HLH和化疗期合并HLH，前者可能与淋巴瘤细胞分泌的细胞因子导致高炎性因子状态有关，而后者是患者在化疗后机体免疫功能受到抑制，在病毒感染和细菌感染的刺激下出现HLH的临床表现[4]。

HLH-94方案和HLH-2004方案仍是目前HLH的一线治疗选择，难治复发的成人HLH患者应用Wang等[5]提出的DEP方案可获得76.2％的总体反应率。在本研究中，不同治疗方案的生存结局有差异，CHOP/CHOP-like方案组患者3年OS较好，而HLH-94方案和HLH-2004方案并未显示生存优势。不同的HLH治疗方案在强度上存在一定的差异，由于临床上缺乏针对患者预后的分层体系，无法形成个体化治疗标准，因此探索成人HLH的预后因素尤为重要。

HLH患者的Fer水平可伴随机体巨噬细胞的增殖及活化产生不同程度的变化[4]。Allen等[6]的研究发现Fer>10 000 µg/L在HLH患者的诊断中有较高的特异性和敏感性。Lin等[7]发现Fer升高与HLH的预后同样密切相关。此外有研究表明Fer与促进肝脏炎症有关，其对炎症的作用涉及Fer对NF-κB的影响[8]。本研究中109例（87.9％）患者Fer≥500 µg/L，50例患者Fer≥2 000 µg/L。但我们并未发现Fer对HLH的预后有影响，Fer是否可作为HLH患者预后的特异性标志物还存在争议[9]。

成人HLH进展较快，是一种严重威胁生命的综合征，病死率较高，本研究124例患者中死亡77例，对存活组与死亡组患者的临床表现及实验室检查数据进行统计学分析，结果表明年龄、FIB、PLT和ALB的差异有统计学意义。单因素、多因素分析显示性别影响成人HLH患者的预后（*P*<0.001），女性和男性患者的3年OS率分别为48.2％和24.5％。多因素分析结果表明PLT、ALB、ALT和治疗方案也是成人HLH的独立预后因素。我们通过限制立方样条和Maxstat统计量获取PLT和ALB的最佳截断值以寻求影响预后的最佳截点。PLT的最佳截断值为58.5×10^9^/L，低于其正常参考值范围。既往一项研究发现PLT<39.5×10^9^/L是HLH患者30 d生存的独立危险因素[10]。本研究124例患者中99.19％患者的PLT低于正常参考值范围上限，50％的患者PLT低于最佳截断值。国外的一项研究表明，当患者病情恶化时PLT下降，病情缓解时PLT上升，因此PLT的变化可作为HLH病情进展的指征之一[11]。本研究发现死亡组的ALB水平低于存活组，ALB水平低对预后有不良影响，与Parikh等[12]的研究结果一致。本研究基于多因素分析筛选的5个独立危险因素建立了列线图模型，C-index为0.739，显示出良好的区分度，校准曲线也提示该模型预测概率与实际概率吻合度较高，保证了列线图的可靠性和重复性。

综上所述，成人HLH是一种具有高度异质性的临床综合征，由于缺少统一的预后评估体系，在个体化的治疗选择上存在一定困难，有效的危险度分层体系有助于个体化分层治疗。本研究通过分析淮海淋巴瘤工作组多个中心成人HLH的临床资料探索预后影响因素，并建立了基于性别、PLT、ALB、ALT和治疗方案的可视化列线图，有助于指导成人HLH的临床治疗。该模型有待多中心前瞻性临床研究验证。

## References

[1] Buyse S, Teixeira L, Galicier L (2010). Critical care management of patients with hemophagocytic lymphohistiocytosis[J]. Intensive Care Med.

[2] Camp RL, Dolled-Filhart M, Rimm DL (2004). X-tile: a new bio-informatics tool for biomarker assessment and outcome-based cut-point optimization[J]. Clin Cancer Res.

[3] 黄 文秋, 王 旖旎, 王 晶石 (2014). 192例成人噬血细胞淋巴组织细胞增生症患者的临床分析[J]. 中华血液学杂志.

[4] Chuang HC, Lay JD, Chuang SE (2007). Epstein-Barr virus (EBV) latent membrane protein-1 down-regulates tumor necrosis factor-alpha (TNF-alpha) receptor-1 and confers resistance to TNF-alpha-induced apoptosis in T cells: implication for the progression to T-cell lymphoma in EBV-associated hemophagocytic syndrome[J]. Am J Pathol.

[5] Wang Y, Huang W, Hu L (2015). Multicenter study of combination DEP regimen as a salvage therapy for adult refractory hemophagocytic lymphohistiocytosis[J]. Blood.

[6] Allen CE, Yu X, Kozinetz CA (2008). Highly elevated ferritin levels and the diagnosis of hemophagocytic lymphohistiocytosis[J]. Pediatr Blood Cancer.

[7] Lin TF, Ferlic-Stark LL, Allen CE (2011). Rate of decline of ferritin in patients with hemophagocytic lymphohistiocytosis as a prognostic variable for mortality[J]. Pediatr Blood Cancer.

[8] Bloomer SA, Brown KE (2019). Iron-Induced Liver Injury: A Critical Reappraisal[J]. Int J Mol Sci.

[9] Schram AM, Campigotto F, Mullally A (2015). Marked hyperferritinemia does not predict for HLH in the adult population[J]. Blood.

[10] Zhao Y, Lu D, Ma S (2019). Risk factors of early death in adult patients with secondary hemophagocytic lymphohistiocytosis: a single-institution study of 171 Chinese patients[J]. Hematology.

[11] Janka G, Imashuku S, Elinder G (1998). Infection- and malignancy-associated hemophagocytic syndromes. Secondary hemophagocytic lymphohistiocytosis[J]. Hematol Oncol Clin North Am.

[12] Parikh SA, Kapoor P, Letendre L (2014). Prognostic factors and outcomes of adults with hemophagocytic lymphohistiocytosis[J]. Mayo Clin Proc.

